# Hip exoskeleton assistance with machine-learning-based state estimation improves gait kinematics of people with Parkinson’s disease

**DOI:** 10.3389/frobt.2026.1770510

**Published:** 2026-03-09

**Authors:** Keaton L. Scherpereel, Jessica E. Bath, Anna Roumiantseva, Jacob Marks, Doris D. Wang, Patrick W. Franks

**Affiliations:** 1 Skip Innovations, San Francisco, CA, United States; 2 University of California San Francisco Department of Physical Therapy and Rehabilitation Science, San Francisco, CA, United States; 3 University of California San Francisco Department of Neurological Surgery, San Francisco, CA, United States

**Keywords:** biomechanics, exoskeleton, gait analysis, machine learning, Parkinson’s disease, wearable robots

## Abstract

Exoskeleton assistance has the potential to address many gait related symptoms of Parkinson’s disease (PD). However, gait variability, a hallmark of PD, makes designing exoskeleton controllers uniquely challenging. We sought to overcome the challenges that gait variability in PD poses for state estimation by employing machine-learning models for gait-phase estimation within our exoskeleton controller. Using machine-learning-based gait-phase models deployed on a hip exoskeleton (N = 7), we performed a 2-day protocol for people with PD where the first day focused on acclimation to the device and the second focused on evaluating the device by collecting gait metrics. Using 2-min walking tests, we assessed the impact of two different types of fixed torque assistance profiles on spatiotemporal and kinematic gait metrics. We demonstrated significant improvements to hip range-of-motion (8.4%), swing time (4.7%), and peak toe clearance (12.3%) in people with PD when walking with a combined flexion and extension assistance profile as compared to walking without an exoskeleton. Although we saw trends, there were no significant differences from providing only flexion assistance given our sample size. We also demonstrated that participant-specific models reduced gait-phase estimation error by 40%, however, resulting gait metrics were not significantly altered compared to metrics when walking with the generic model. These results demonstrate that ML gait-phase-based control approaches with limited PD-specific data can improve PD gait kinematics, with enhanced accuracy associated with participant-specific data. Ultimately, these results contribute to the goal of assistive exoskeletons in everyday use for people with Parkinson’s disease.

## 1 Introduction

User variability is an important consideration in exoskeleton control that substantially hampers exoskeleton effectiveness, specifically due to the importance of precise timing and magnitude of assistance (Zhang et al., 2017; Ding et al., 2018; Franks et al., 2021). This variability hampers performance in two distinct areas: user state estimation (Lee et al., 2024) and assistance profile determination (Slade et al., 2022). Recent advances in machine learning (ML) for exoskeleton control have shown great potential to overcome challenges due to user variability in both areas through improved state estimation (Shepherd et al., 2022; Lee et al., 2024; Molinaro et al., 2024) and through estimating user-desired assistance profiles (Lee et al., 2023). However, the majority of these studies focus on young, able-bodied users, while the potential impact is likely greater for clinical populations where heterogeneity in disease presentation could render adaptable and personalized assistance even more valuable for overcoming gait impairments. This is particularly relevant for Parkinson’s disease (PD), where greater kinematic variability and individualized disease manifestation differences are a hallmark of the disease (Hausdorff, 2009; Ryden and Lewis, 2019). Our work seeks to address the state estimation portion of this gap by assessing the application and utility of ML gait-phase-based exoskeleton control techniques in people with PD exhibiting gait impairments.

Over 10 million people worldwide live with PD, and its prevalence continues to rise (Peng et al., 2025). While many of the symptoms of PD can be managed with medication and surgical therapies, there is currently no cure for the underlying condition (Kobylecki, 2020). A prominent and particularly detrimental motor symptom of PD is gait impairment, which worsens with disease progression (Mirelman et al., 2019). People with PD often exhibit decreased walking speed, stride length, and hip range of motion, and increased stride frequency (Zanardi et al., 2021) as well as decreased toe clearance (Alcock et al., 2016, Alcock et al., 2018). These gait related symptoms significantly increase fall risk (Creaby and Cole, 2018), decrease overall mobility, and negatively impact quality of life (García et al., 2019).

Current treatment options to address PD-related gait impairments include medications such as levodopa, neuromodulation therapies such as deep brain stimulation, and physical therapy (Mirelman et al., 2019; Smith et al., 2021). Medication is generally used as a first-line treatment for overall PD symptoms; however, it often produces variable effects on axial symptoms such as gait impairment (Curtze et al., 2015). Deep brain stimulation can provide significant gait benefits, but not all patients are eligible, and it can even cause worsening of gait and balance (Daalen et al., 2025). Physical therapy and/or targeted exercise can be effective for improving multiple aspects of PD gait without the potential side effects of pharmacological or surgical strategies (Radder et al., 2017; Mirelman et al., 2019). However, consistency and duration are critical factors that influence the effectiveness and lasting benefit of training (Mak et al., 2017). This type of personalized care also places a burden on the healthcare system (Smith et al., 2021) while offering variable gait improvements, especially for long-term outcomes (Comelia et al., 1994; Ni et al., 2018) and for those with freezing of gait (Gilat et al., 2021).

Recent advances in wearable exoskeletons offer a new and potentially synergistic path to realizing the benefits of non-invasive, non-pharmacological mobility assistance in one’s daily life. For PD, a recent N = 1 study demonstrated that a hip exoskeleton providing only flexion assistance can reduce freezing of gait (a prevalent gait issue associated with progressed PD) (Kim et al., 2024). Gait training assisted by a hip exoskeleton has also demonstrated improvements in gait kinematics both immediately upon donning and after several training sessions, although with limited retention 1 month after training (Kawashima et al., 2022; Otlet et al., 2023). However, none of these studies have benefited from the state estimator performance benefits of ML techniques. This is especially relevant for PD, where motor fluctuations across medication states can differentially impact gait functions, causing fluctuations in gait impairments such as shuffling or freezing, which could make generic controllers and able-body-trained models much less relevant (Kang et al., 2025). Additionally, while previous work has focused on rehabilitation applications, advances in lightweight actuators and comfortable interfaces enable devices to be used beyond rehabilitation in everyday life.

With this objective in mind, our study took advantage of ML gait-phase estimation techniques coupled with fixed torque profiles to explore two specific hypotheses vital for assessing the feasibility and efficacy of a regular-use assistive exoskeleton for people with PD exhibiting gait impairments. First, we hypothesized that using hip exoskeleton assistance with ML based gait-phase estimation will improve PD gait as measured by increased hip range of motion (ROM) and increased walking speed compared to walking without an exoskeleton. Second, we hypothesized that participant-specific gait-phase models will be more accurate than generic models and result in improved gait metrics such as hip ROM and walking speed.

## 2 Methods

**FIGURE 1:**
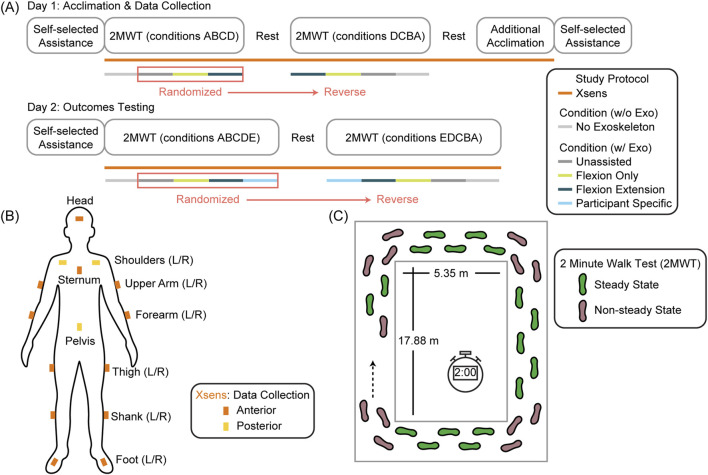
Study protocol. **(A)** Our study involved two separate days with the first focused on acclimation and the second focused on outcome evaluation. Users performed 2 minute walk tests (2MWT) during multiple exoskeleton conditions including walking without the exoskeleton while gait metrics were collected using Xsens **(B, C)** Gait metrics were analyzed for the steady-state, straight-line walking segments only.

To analyze the impact of our autonomous robotic hip exoskeleton on PD gait, we collected gait metrics from seven people with PD while they participated in a 2-day experimental protocol. All participants were tested while on PD medication, if applicable. The first day was focused on acclimation to the device and collecting preliminary biomechanics data. The second day focused on evaluating the impact of our hip exoskeleton during a series of 2-min walking tests (2MWT) where we compared different controllers on the device to two baselines: wearing the device without assistance and not wearing the device at all. During both days we used an inertial measurement unit (IMU) motion capture system (Xsens MVN, Movella) with 15 sensors to collect gait kinematics. This approach is summarized in Figure 1.

### 2.1 Participants

We set out to recruit ten participants by first performing a virtual screening in which we obtained informed consent and then assessed preliminary eligibility using the Mini-Montreal Cognitive Assessment (MoCA) exam and Movement Disorder Society Unified Parkinson’s Disease Rating Scale, motor subscale (MDS-UPDRS-III) over secure videoconference. This assessment helped to determine whether the participant would likely meet the study inclusion criteria consisting of a diagnosis of idiopathic PD, MoCA score >20, the presence of PD-related gait issues, no history of cardiovascular events within the last 12 months, and projected ability to complete our intended protocol, including the walking tests. At the first study visit, the full MoCA and MDS-UPDRS-III tests were performed to verify participant eligibility if those assessments had not been performed within the last 6 months. Participants provided informed written consent under Salus IRB-23243. All tests and measures were performed by qualified and trained study personnel.

Although we enrolled ten participants, three participants did not complete the protocol as intended and thus were removed from the aggregate analysis for a variety of reasons. Specifically, one participant had severe freezing of gait on day two which prevented continuous walking. Due to a device malfunction, one participant only received actuation on one side and thus was not included in aggregate results. The final participant excluded from analysis chose device assistance below our minimum threshold and thus could not be compared across controller conditions. Thus, our final aggregate analyses consisted of seven participants, including six males and one female (Table 1). Participants were tested while ON-medication, if applicable. Although four of our seven participants reported freezing of gait as a symptom, none of the participants experienced noticeable freezing of gait during the 2MWTs that we performed. A gender skew toward males is reflective of the overall gender differences in the PD population (Miller and Cronin-Golomb, 2010).

**TABLE 1:** Participant demographic and clinical metrics.

Participant ID	Age range	Gender	H&Y stage	MDS-UPDRS II	MDS-UPDRS III (on-med)	Clinical treatment	Disease duration (years since diagnosis)
PD1	70–79	F	2	12	25	Medication	2
PD2	60–69	M	2	11	44	Medication	9
PD3	60–69	M	2	12	29	Medication, DBS	8
PD4	70–79	M	2	13	31	Medication, DBS	17
PD5	70–79	M	2	10	37	Medication, DBS	>10
PD6	70–79	M	2	21	36*	None	5
PD7	70–79	M	2	14	45	Medication	6

Abbreviations: Hoehn & Yahr (H&Y), Movement Disorder Society Unified Parkinson’s Disease Rating Scale (MDS-UPDRS), Deep Brain Stimulation (DBS), *Off medication due to current treatment status.

### 2.2 Study protocol

At the beginning of both days one and two, participants were first fitted with the device and then performed an unstructured walking bout while selecting their preferred assistance level for each of the two powered device controllers (flexion-only assistance and flexion-extension assistance). Assistance level is a scalar value applied to open-loop current commands to adjust the magnitude of assistance. During this time, we gradually increased the assistance level of the device from a preset baseline while asking the participant for continuous feedback on whether the assistance was feeling comfortable while they walked at their preferred comfortable walking speed. Once assistance became uncomfortable, we decreased the assistance back to a comfortable level and then set that level for the future conditions with that controller. In total, the user walked with assistance for approximately 2–3 min during this preferred assistance assessment period. Once the assistance level was set for both controllers, the device was doffed and the Xsens IMU sensors were placed on the participants’ body and calibrated (Figure 1B).

For both day one and two, each participant performed a series of 2MWTs while completing laps around a square hallway (Figure 1C) at a comfortable walking speed (Lakmazaheri et al., 2024) while the distance walked was continuously measured. The 2MWT was selected to enable multiple testing conditions while minimizing accumulated participant fatigue, a potential barrier for testing in people with PD with potentially impaired walking endurance. The 2MWT is a valid and reliable method to capture walking capacity in people with PD and is correlated with walking tests of longer duration (Witherspoon et al., 2019; Johansson et al., 2025). We performed these tests in a reverse ladder design (ABCD-DCBA) to minimize any confounding effects of fatigue and muscle stiffness over the course of the session. For day one, we performed the walking test once without the device and then three times with the device using a random permutation of three controllers: flexion-only assistance, flexion-extension assistance, and unassisted. For day two, we performed the same series of 2MWTs, but with one extra condition using the flexion-extension controller based on an ML model fine-tuned with data collected from that participant during day one (ABCDE-EDCBA), termed the “participant specific” condition. This condition was randomized along with the other three device-donned conditions (Figure 1A). Other than the experimenter controlling the exoskeleton, personnel and participants were blinded to the three or four controller conditions (the no exoskeleton condition could not be blinded). Backdriving the motor during the unassisted device condition still creates noise, thus it was not obvious visually or auditorially which exoskeleton condition was being used. After each 2MWT, we queried the participant to provide their rate of preserved exertion (RPE) score using the Borg Category-Ratio10 scale (Johansson et al., 2025) and a single Activities-Specific Balance Confidence (ABC)-style score for their walking during the previous condition (“How confident are you that you will not lose your balance or become unsteady?”). Due to medication schedules, several participants required a medication break during the experiment time, which was timed to coincide with the enforced break in the middle of the reverse ladder design to avoid changing medication states within a side of the ladder that would affect the comparison of controllers.

On day one, participants also completed some additional walking and turning tasks at the end of the session while in the device to become more acclimated to it and collect more walking data for the participant-specific model training. In total, the combination of these additional day one tasks in the exoskeleton totalled another 3–5 min with the device providing flexion-extension assistance. After these activities, we repeated the search for self-selected assistance levels in the same way that we performed the search at the beginning of the day to verify a similar level of comfort with the device.

At the very end of each day, we gave participants a survey to obtain their qualitative feedback about the performance of the device and their perceptions of its impact. We asked two main qualitative questions about participants’ perception of the device during the study: 1. “How did it feel to walk in the device today? On a scale from 1 Much harder than without it to 5 Much easier than without it” and 2. “Which of these did the device feel helpful for today? Please select all that apply and/or leave this blank if the device did not feel helpful: Feeling less fatigued, Having less freezing episodes, Feeling more balanced, Taking longer steps, Walking more regularly/symmetrically, Walking faster, Picking up your feet, Walking more confidently, Being more upright, Other”. All results are reported for the survey from the second day of testing.

### 2.3 Device and controller design

Our autonomous hip exoskeleton provides sagittal plane assistance at the hips using custom actuators (Skip Innovations, San Francisco, CA) mounted coaxially with the hip joint. Although our actuators can provide torques peaking at 30 Nm, the comfort of the structure and coupling to the body limited our assistance to ∼10 Nm. The exoskeleton consists of a waist-belt fixed to a carbon fiber and 3D printed structure that supports the actuators. Thigh struts extend from the actuators and attach to the thighs using webbing straps. Additional thigh straps attach directly to the pelvis structure to better couple the motors to the body and a rear strap minimizes twisting forces. A further additional waist strap and suspenders were used with some participants to aid with fit around the midsection if sufficient coupling to the body was impossible without them. The total weight of the device is 2.3 kg. Control and communication is facilitated by a raspberry pi (Raspberry Pi, Cambridge, England) while sensing consists of four 6-axis IMUs mounted along the thigh struts with two on each leg and encoders for joint angle and velocity at each motor. For data collection purposes and not for control, IEE insoles (IEE, Luxembourg) were worn inside the participants’ shoes during testing to collect vertical ground reaction force estimates and motors were strain gauged to provide estimates of user experienced torques.

**FIGURE 2:**
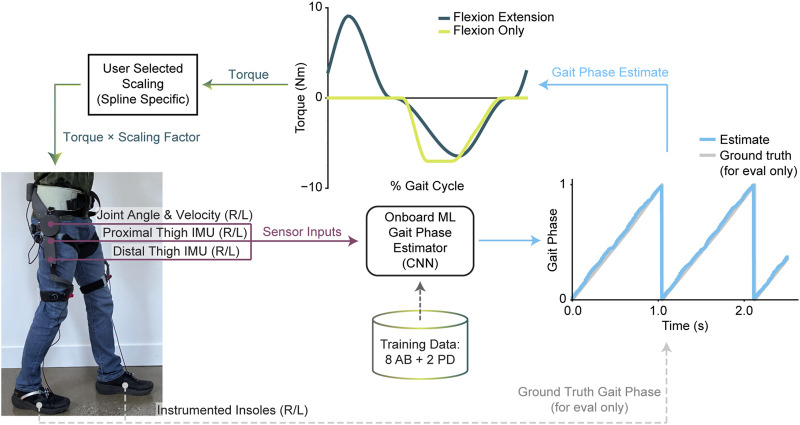
Exoskeleton controller design. Our autonomous hip exoskeleton measures movement using four thigh mounted inertial measurement units (IMUs) and two hip encoders. These sensor inputs are used to estimate gait phase using an ML estimator trained with both able-bodied and PD data. Estimated gait phase is used to determine assistance torque based on predefined splines and then scaled by a user-selected scaling factor specific to each assistance profile.

To control our autonomous hip exoskeleton, we trained a machine learning (ML) model to estimate gait phase during real-time operation. ML inference was run on the raspberry pi at 100 Hz. To translate from gait phase to desired assistance torque, we used two different types of controllers defined by eight nodes connected with piecewise cubic Hermite interpolating polynomials. The first controller provided flexion and extension assistance where the nodes were based on human-in-the-loop optimal energetic splines (Franks et al., 2021). The second controller provided flexion-only assistance where the nodes were previously tuned manually based on user preference. The commands from these splines were scaled linearly to open loop current commands based on the user’s desired assistance level for that specific controller type. The scalar value (e.g., 0.8x) was applied to the entire spline. The control loop and hardware is depicted in Figure 2.

### 2.4 Model training

To train the generic ML model, we collected training data from eight young, able-bodied users performing level overground walking as well as some stairs and two PD users (1 male H&Y 2 & 1 female H&Y 3) performing level overground walking. Training data included data collected while the device was unactuated, providing flexion-only assistance, and providing flexion-extension assistance. Insole estimates of vertical ground reaction force were used to determine heel strike and toe off based on force thresholds set from the lowest quartile of force data. These were converted to ground-truth gait-phase labels with a linear fit from 0% to 60% representing stance phase (heel strike to toe off) and a linear fit from 60% to 100% representing swing phase (toe off to the following heel strike). Labels were examined by hand to verify integrity.

Sensor inputs to the model consisted of 3-axis acceleration and 3-axis gyroscope measurements from the four thigh-mounted IMUs as well as joint position and velocity from the encoders at each hip joint. The model was fed these features in a window of 10 samples spanning 200 ms where each time series feature entry is the average of 20 ms of data. The output of the model was an estimate of gait phase using a Cartesian version of gait phase (x and y value) to preserve the cyclic nature of gait phase during ML training (Kang et al., 2021; Lee et al., 2024). This Cartesian version was then converted to percent gait phase using a polar angle conversion. The model architecture used three 1-D convolutional layers followed by one final output dense layer to estimate gait phase inspired by Shepherd et al. (Shepherd et al., 2022). We trained the model for a set number of 15 epochs with data from both the left and right leg by mirroring the data for the contralateral leg (Molinaro et al., 2023; Scherpereel et al., 2024). At inference time we performed the same mirroring operation and thus ran the same model with a batch size of two to get left and right estimates of gait phase.

To train participant-specific models for day two, we froze the convolutional layers of the generic model, dropped the learning rate, and used experimenter-labeled data collected with the device on day one to retrain the dense layer of the model, a common fine-tuning transfer learning approach (Xu et al., 2021; Lv et al., 2022). We divided the learning rate by five and limited the retraining to ten epochs. We chose ten epochs based on an initial validation where we saw improvement level out on held-out 2MWTs for a PD participant. We had to remove two additional participants from the participant-specific model results (N = 5) that were otherwise in the aggregate results; one was in the original training data (from a pilot session several months earlier) and the other experienced an exoskeleton clock rate issue limiting our ability to get an accurate label to on-board estimate comparison.

### 2.5 Data analysis

All gait analysis was performed on data collected with the Xsens IMU sensors to ensure the same analysis pipeline regardless of whether the user was wearing the device. Measurements of the user’s anthropometry were used to scale a human skeleton within the Xsens toolbox in accordance with the manufacturer’s protocol.

The active portion of the 2MWT was determined by the initiation of gait detected by center of mass (COM) velocity. The initial 2 seconds of data were removed from analysis to remove initial acceleration. Gait cycles were then segmented by kinematic foot trajectory (using foot velocity below a threshold for heel strike and vertical toe velocity above a threshold for toe off) and then examined by hand. Turning sections were removed by removing strides where the COM direction changed more than a threshold (11°) from the previous step. This procedure ensured that the metrics analyzed were for steady state walking (Figure 1C) (Romijnders et al., 2021).

Gait metrics were extracted for each valid stride and then averaged across individual 2MWTs. Metrics were then averaged across the two tests with the same exoskeleton condition. Finally, the metrics were averaged across right and left legs. The gait metrics are defined as follows: hip ROM (the maximum hip angle minus the minimum hip angle over a stride), stride length (the projected change in position vector for the foot over a stride onto the COM position change over that stride to ensure that the metric is always measuring distance in the direction of motion), swing time (the time from toe off to heel strike), stride frequency (the inverse of the stride time), and peak toe clearance (the maximum vertical toe displacement during late swing phase).

These metrics cover the two main categories of metrics used to evaluate PD gait that show significant alterations in PD: spatiotemporal and kinematic measures (Bonacina et al., 2024). For our central hypothesis in this study we chose a metric from each of these categories, namely, walking speed as a spatiotemporal characteristic, and hip ROM as a kinematic metric. Walking speed is significantly reduced in PD (Zanardi et al., 2021) and exoskeletons can improve walking speed in older adults (Lakmazaheri et al., 2024). While many studies purely focus on spatiotemporal characteristics, kinematic metrics can more effectively differentiate small changes in gait and also are often less linked to higher level cognitive decisions (Albani et al., 2014). Hip ROM is widely used when kinematic analysis is performed, is most directly related to the intervention of a hip exoskeleton, and shows a substantial decrease across the PD population as a whole (Zanardi et al., 2021).

### 2.6 Statistics

Statistics were run in python using the pingouin python package. For results across multiple controller types (no exoskeleton, unassisted, flexion-extension, flexion-only), a one-way repeated measures analysis of variance test (ANOVA) across participants was used with a significance level of alpha = 0.05. The dependent variable was the gait metric outcome of interest and the independent variable was the controller type. The participant was the random effect. If a significant effect was observed, we followed up with multiple planned comparisons with Bonferroni correction. Prior to examining the results, we planned to compare only the controller conditions against no exoskeleton to maintain the power of the test (thus only performing three comparisons rather than all six). For results across two controller types (generic versus the participant-specific model), a paired t-test was used with significance level of alpha = 0.05 with participant as the random effect.

## 3 Results

**FIGURE 3:**
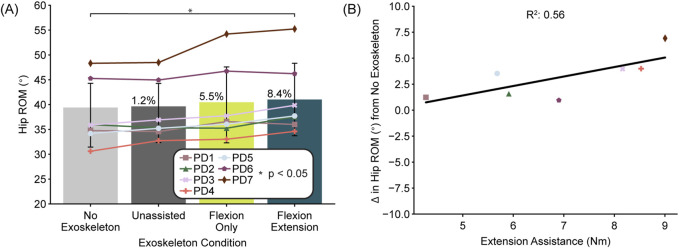
Hip range of motion (ROM) by exoskeleton condition. **(A)** Average hip ROM for each exoskeleton condition is presented across participants (N = 7). Bars represent the average, error bars represent the standard deviation, and individual participants are indicated by specific symbols. Asterisks indicate statistical significance (p < 0.05) determined by planned comparisons with Bonferroni correction. Percentages represent the percentage difference between the condition average and the no exoskeleton condition. **(B)** Magnitude of extension assistance versus the change in hip ROM is presented for each participant in the flexion extension condition. The line represents the line of best fit across participants with the R^2^ value of the fit indicated above.

Across participants (N = 7), exoskeleton controller type had a significant effect on hip ROM (one-way ANOVA, F = 10.01, df = 3, p < 0.01) (Figure 3A). Our post-hoc multiple comparisons tests revealed a significant increase in hip ROM when using the flexion-extension controller compared to no exoskeleton (p = 0.02) of 8.4% (3.2°). When using the flexion-only controller, we did not see a significant increase in hip ROM compared to no exoskeleton (p = 0.09) even though the change was 5.5% (2.1°). To further explore this finding, we compared the relationship between the assistance magnitude and hip ROM and found a positive correlation between the change in hip ROM relative to no exoskeleton and hip extension assistance magnitude (R^2^ = 0.56) (Figure 3B).

**FIGURE 4:**
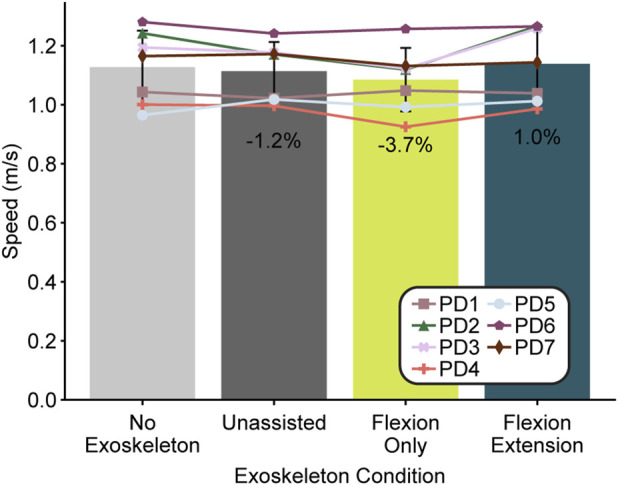
Walking speed by exoskeleton condition. The average walking speed across participants (N = 7) for each condition is presented by the bars with error bars representing the standard deviation and each participant marked by a specific symbol. Percentages show the percentage difference between the condition average and the no exoskeleton condition.

Although our one-way ANOVA across participants did indicate a significant difference in walking speed based on controller type (F = 3.47, df = 3, p = 0.04), the post hoc comparisons could not detect a difference between conditions (Figure 4). Despite this fact, six of the seven participants reported feeling like the device helped them walk faster.

**FIGURE 5:**
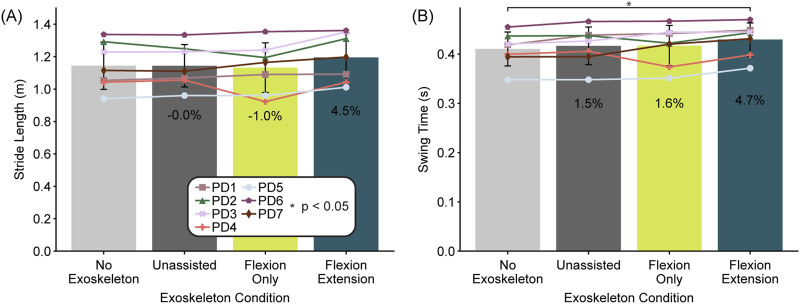
Stride length and swing time by exoskeleton condition. Bars represent the average across participants (N = 7) for stride length **(A)** and swing time **(B)** separated by condition. Error bars represent the standard deviation. Individual participants are marked with a specific symbol and percentages indicate the average difference compared to no exoskeleton. Asterisks represent statistical significance (p < 0.05) determined by planned comparisons with Bonferroni correction.

Stride length changed with controller type (one-way ANOVA, F = 4.28, df = 3, p = 0.02), but although the flexion-extension controller increased stride length by 4.5% (0.051 m) compared to no exoskeleton, it was not significant (p = 0.06) (Figure 5). Six out of seven participants reported feeling like the device helped them take longer strides. Swing time also changed with controllers (one-way ANOVA, F = 4.15, df = 3, p = 0.02) increasing with the flexion-extension controller compared to no exoskeleton (p = 0.03) by 4.7% (0.02 s) (Figure 5).

**FIGURE 6:**
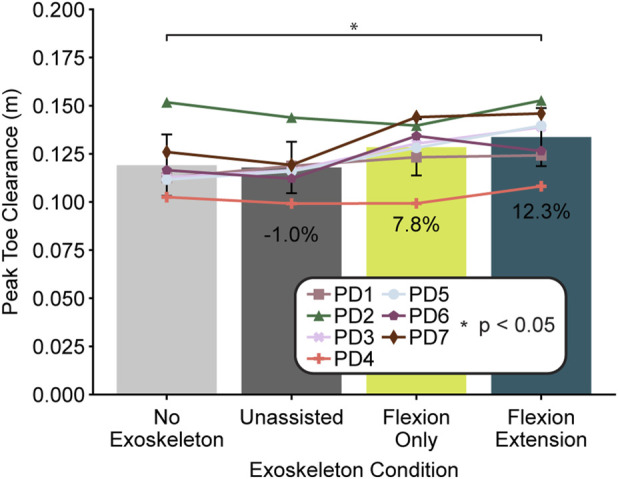
Peak toe clearance during late swing phase by exoskeleton condition. Bars represent the across-participant average (N = 7) per condition and error bars represent the standard deviation. Individual participants are marked with a specific symbol. Percentages indicate the percent difference in averages as compared to the no exoskeleton condition. Asterisks indicate statistical significance (p < 0.05) assessed using planned comparisons with Bonferroni correction.

To further assess the kinematic changes due to walking with the exoskeleton, we also examined changes in peak toe clearance during late swing phase. Peak toe clearance changed with controller type (one-way ANOVA, F = 9.07, df = 3, p < 0.01) with the flexion-extension controller significantly increasing peak toe clearance (p = 0.03) by 12.3% (0.015 m) (Figure 6). Six out of seven participants reported feeling like the device helped them to pick up their feet.

Although our post-experiment surveys showed that the majority of users found it easier to walk with the device (4 users said it was “somewhat easier,” 1 said “much easier,” and 2 said “about the same”), individual RPE scores showed no consistent trends in performance (RPE: No Exoskeleton 1.2 ± 0.8, Unassisted 1.5 ± 0.9, Flexion Only 1.4 ± 1.0, Flexion Extension 1.5 ± 1.0). ABC type scores across conditions also did not show any trends (ABC: No Exoskeleton 92 ± 12, Unassisted 91 ± 10, Flexion Only 91 ± 11, Flexion Extension 93 ± 9) and four out of seven participants said that the exoskeleton helped them to feel more balanced. No falls or adverse events occurred during the study. All users reported some perceived benefits from our list while wearing the device. The exact responses for each participant after the second day of testing are included in the associated dataset.

**FIGURE 7:**
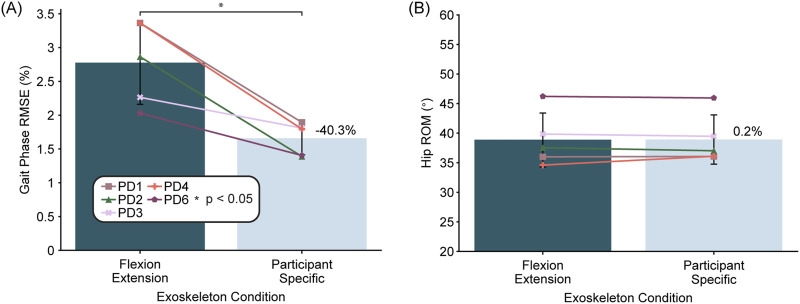
Comparison between the generic gait-phase estimator and the participant-specific estimator. Bars indicate the across participant average (N = 5) for gait-phase model root mean squared error (RMSE) **(A)** and hip range of motion (ROM) **(B)**. Error bars represent the standard deviation across participants and the percentages indicate the percent difference between the participant-specific model compared to the generic model. Asterisks indicate statistical significance (p < 0.05) as determined by paired t-tests.

When comparing the flexion-extension controller with the participant-specific gait-phase estimator versus with the generic gait-phase estimator (N = 5), we found that the participant specific gait-phase estimator significantly decreased estimation error (p < 0.01), dropping it by 40% from an average of 2.8% phase root mean squared error (RMSE) to 1.7% phase RMSE (Figure 7A). However, the hip ROM did not change significantly when using the participant-specific models (Figure 7B) and neither did the other gait metrics.

## 4 Discussion

These results indicate that our autonomous hip exoskeleton was able to alter gait kinematics in the direction of healthy, age-matched controls for people with PD, specifically by increasing hip ROM (increase of 3.2° versus PD population decrease of 5.3°) (Zanardi et al., 2021). The correlation between increased hip ROM and exoskeleton assistance indicates that more benefit could be accrued if greater assistance could be provided. Because we allowed users to self-select the assistance level, we were limited by their perceptions of the device and its comfort. Giving users more time to acclimate to the device is likely to improve their ability to accept and utilize assistance torque (Ingraham et al., 2022). Also, improving the human-exoskeleton interface to better couple to the body, especially for non-symmetric torques (unequal flexion and extension), may increase user comfort with higher torques with the potential drawback of additional weight and discomfort for longer-term use.

Contrary to our hypothesis, walking speed did not increase even though other gait metrics did change. While perhaps surprising given that stride length increased, the observation of a 3% decrease in stride frequency may counteract the impact of stride length differences. Increased stride frequency is associated with PD gait, with our results suggesting a return toward more physiologically healthy gait patterns (Zanardi et al., 2021). The lack of change in walking speed was also perhaps due to the instruction to walk at a comfortable walking pace which users continued to do across conditions controlled by higher-level executive functions (Albani et al., 2014). Although walking speed did not change, we did see changes in sub-phase durations, specifically a significant increase in swing time in the direction of healthy controls (increase of 4.7% versus PD population decrease of 1.8%) (Zanardi et al., 2021). While not directly comparable to other studies, our stride length change of 0.05 m is similar to the initial device changes of a previous study (Kawashima et al., 2022) and our hip ROM change of 8.4% is similar to the effects of a month of exoskeleton gait training in another (Otlet et al., 2023). By including the unassisted condition and verifying no significant differences across gait metrics when compared to the no exoskeleton condition, we can confirm there were no significant placebo effects from wearing a new device.

Our results from peak toe clearance similarly indicate kinematic changes in the direction of healthy, age-matched controls (increase of 0.015 m versus early stage PD decrease of 0.011 m) (Alcock et al., 2016, Alcock et al., 2018; Ferreira et al., 2019). This metric has also been shown to relate to spatiotemporal variables and foot kinematics, such as increased stride length and decreased stride frequency (Cho et al., 2010; Alcock et al., 2018), thus increased peak toe clearance further corroborates our other results. Although foot clearance-related metrics are associated with fall risk, the connection is not always direct (Barrett et al., 2010; Avalos and Rosenblatt, 2024), so further longitudinal studies are needed to determine any potential changes in participants’ fall risk. Toe clearance results must be analyzed critically given that they were derived from IMU measurements; however, previous studies have also reported toe displacement metrics directly from Xsens (Fonseca et al., 2021) and non-proprietary algorithms have reported good correlation to optical motion capture (Mariani et al., 2012; Hamacher et al., 2014). Additionally, in this study, the condition comparison results are an intra-participant, intra-day comparison collected using a reverse ladder study design. Thus, it is specifically designed to handle sensor drift.

The lack of consistent trends in RPE across conditions is not surprising given the participants’ relatively low overall RPE scores (likely due to the provided instructions to walk at a comfortable pace rather than to walk “as far as possible” (Johansson et al., 2025)) and large standard deviations nearly as large as the value itself. RPE is also less sensitive in people with PD compared to able-bodied individuals (Penko et al., 2017). Consistent one-question ABC ratings observed across conditions and the fact that more than half of the users reported feeling more balanced in the device are a potentially positive outcome since, at a minimum, there were no measurable detrimental effects from exoskeleton assistance on participants’ confidence in their balance. Allowing users to self-select the torque assistance also likely contributed to this effect.

In general, user perception tracked with quantitative metrics. However, in certain cases it did not. For the balance questions, one important consideration is that the ABC-style question was asked after each 2MWT. This smaller time scale could perhaps be more accurate, but it also means the same question is asked many times (up to ten times) which could lead to feedback fatigue. The final qualitative survey question was asked as an overall question, thus on a longer timescale, but combining all feelings from the entire day. For the walking speed question, while it is possible that the altered sub-phase durations impacted perception, it is more likely that participants’ perception of speed was simply incorrect due to known perception impairment in PD (Halperin et al., 2021).

Although we did not directly analyze the difference between the flexion-only and flexion extension controllers, all of the metrics indicated more changes in the direction of healthy age-matched controls with the flexion extension controller compared to the flexion-only controller. From an energetics viewpoint, we expect larger changes from including flexion plus extension assistance because it increases the amount of time in which the device can inject positive energy to the user. We also found no significant differences between the flexion-only controller and no exoskeleton across any of the outcome metrics. Previous work has shown that flexion-only controllers can have a beneficial impact on freezing of gait for people with PD (Kim et al., 2024) and thus it would be reasonable to expect a difference in kinematics as well. Although we found no significant differences between the flexion-only controller and no exoskeleton, it is possible that the lack of detection is due to sample size rather than a lack of efficacy of flexion-only assistance. Given the additional benefit of extension assistance in addition to flexion, future work could consider flexion-extension assistance for addressing freezing of gait.

Examining participant-specific models revealed positive outcomes for estimation accuracy, decreasing the error by more than a third. This is particularly relevant for PD because of the inherent variability in PD gait (Del Din et al., 2016) and specifically within this study due to the treatment differences across participants. Despite day-to-day variability in PD, data from a previous day was still relevant for decreasing estimation error. However, we did not see this translate to the participants’ gait metrics. While the percentage error decrease is substantial, the generic gait-phase estimator performed well on our predictable walking task. In a previous study (Franks et al., 2021), the optimized hip extension torque gait-phase difference was 0.6% across users and flexion torque difference was 1%, whereas our improvement from the participant specific model was 1.1%. Thus, even though the percent change was substantial, the efficacy of the controller is likely dominated by other effects such that the additional timing accuracy did not contribute enough to visualize. These participant-specific fine-tuning results indicate that participant-specific models may be worth pursuing for other estimates, such as more end-to-end AI-based controls approaches (Molinaro et al., 2024), where improved estimation performance may be more tied to user outcomes than it was for gait-phase-based spline control on level ground in our study.

The changes observed in this study were based on a relatively short period of time in the exoskeleton separated across several days, however research has shown that adaptation time even for able-bodied users requires significantly more time in the device (Poggensee and Collins, 2021). Also, research indicates that those with gait impairments may take longer to adapt (Lerner et al., 2019). Thus, we anticipate that greater benefits could be accrued with longer training times or with in-home use.

These results indicate that exoskeleton assistance may be able to complement the current pharmacological and neurological approaches to treating gait impairments in people with PD. As exoskeletons become more deployable in naturalistic settings, these results indicate that assistive exoskeletons may facilitate a greater maintenance of functional mobility over time in people with PD, which may also help to slow disease progression.

### 4.1 Limitations

There are several limitations in this study. First, our limited participant count limits further exploration of small potential differences between conditions. Second, we only studied participants with mild gait impairment (all participants were at H&Y stage of 2) who could complete our protocol. Further exploration is needed to analyze the impact of an exoskeleton on users with more severe gait impairments, including freezing of gait. Third, our use of IMU-based motion capture without force plates versus optical motion capture with force plates limited us to only kinematic and spatiotemporal measures without kinetic comparisons and increased our reliance on relative rather than absolute changes for global displacement metrics. Fourth, we did not optimize the ML model architecture for gait-phase estimation so further optimization may decrease estimation error.

### 4.2 Conclusion

Our results demonstrate that an ML gait-phase-based control system with an autonomous hip exoskeleton can improve gait kinematics in people with PD during level overground walking. We saw significant increases in hip ROM, swing time, and peak toe clearance all in the positive direction of healthy controls while walking with flexion and extension assistance. While these represent the immediate benefits from donning and using the device, benefits could potentially increase as a user becomes more familiar with the device. Furthermore, we demonstrated that participant-specific model fine-tuning can decrease model error. The benefits of user-specific model fine-tuning for people with PD and gait impairment potentially indicate that other ML-based methods could be extended to clinical applications with a similar fine-tuning approach. This could unlock other control approaches that enable users to use devices in even more unstructured environments during in-community use. These results demonstrate the efficacy of an exoskeleton in improving gait quality for people with PD and provides insights into the design of a flexible control system that can handle the variability of PD presentation during regular exoskeleton use.

## Data Availability

The human movement data collected in this study along with the processed data and qualitative survey data used for these results is available at the Zenodo repository under doi: 10.5281/zenodo.17970178.
